# Tuberculosis control strategies to reach the 2035 global targets in China: the role of changing demographics and reactivation disease

**DOI:** 10.1186/s12916-015-0341-4

**Published:** 2015-04-21

**Authors:** Grace H Huynh, Daniel J Klein, Daniel P Chin, Bradley G Wagner, Philip A Eckhoff, Renzhong Liu, Lixia Wang

**Affiliations:** Institute for Disease Modeling, 1555 132nd Ave NE, Bellevue, WA 98005 USA; China Office, The Bill & Melinda Gates Foundation, Beijing, 100027 China; Chinese Center for Disease Control and Prevention, Beijing, 102206 China

**Keywords:** Tuberculosis, China, Global targets, Latent reservoir, Reactivation, DOTS

## Abstract

**Background:**

In the last 20 years, China ramped up a DOTS (directly observed treatment, short-course)-based tuberculosis (TB) control program with 80% population coverage, achieving the 2015 Millennium Development Goal of a 50% reduction in TB prevalence and mortality. Recently, the World Health Organization developed the End TB Strategy, with an overall goal of a 90% reduction in TB incidence and a 95% reduction in TB deaths from 2015–2035. As the TB burden shifts to older individuals and China’s overall population ages, it is unclear if maintaining the current DOTS strategy will be sufficient for China to reach the global targets.

**Methods:**

We developed an individual-based computational model of TB transmission, implementing realistic age demographics and fitting to country-level data of age-dependent prevalence over time. We explored the trajectory of TB burden if the DOTS strategy is maintained or if new interventions are introduced using currently available and soon-to-be-available tools. These interventions include increasing population coverage of DOTS, reducing time to treatment, increasing treatment success, and active case finding among elders > 65 years old. We also considered preventative therapy in latently infected elders, a strategy limited by resource constraints and the risk of adverse events.

**Results:**

Maintenance of the DOTS strategy reduces TB incidence and mortality by 42% (95% credible interval, 27-59%) and 41% (5-64%), respectively, between 2015 and 2035. A combination of all feasible interventions nears the 2035 mortality target, reducing TB incidence and mortality by 59% (50-76%) and 83% (73-94%). Addition of preventative therapy for elders would enable China to nearly reach both the incidence and mortality targets, reducing incidence and mortality by 84% (78-93%) and 92% (86-98%).

**Conclusions:**

The current decline in incidence is driven by two factors: maintaining a low level of new infections in young individuals and the aging out of older latently infected individuals who contribute incidence due to reactivation disease. While further reducing the level of new infections has a modest effect on burden, interventions that limit reactivation have a greater impact on TB burden. Tools that make preventative therapy more feasible on a large scale and in elders will help China achieve the global targets.

**Electronic supplementary material:**

The online version of this article (doi:10.1186/s12916-015-0341-4) contains supplementary material, which is available to authorized users.

## Background

Significant progress in tuberculosis (TB) control has been achieved worldwide over the last two decades. Global TB mortality has fallen by 45%, and TB incidence is declining [1]. Recently, the World Health Organization (WHO) established an ambitious post-2015 global strategy, the End TB Strategy [2]. This strategy outlines a 2025 milestone of 50% reduction in incidence and 75% reduction in mortality, and an overall 2035 target of 90% reduction in incidence and 95% reduction in mortality. In order to reach these targets, countries will likely need to redouble their TB control efforts and perhaps adopt new TB control strategies [3].

Between 1992 and 2012, China made impressive progress in TB control. Prior to 1992, most TB patients were treated in private hospitals, where patients typically received low-quality care - improper treatment was widespread, and only approximately 20% of patients had supervised TB treatment. In addition, nearly 50% experienced interrupted or shortened treatment and there was little follow-up of patients who dropped out or relapsed after a treatment episode [4-7]. Starting in 1992, China ramped up a high-quality directly observed treatment, short-course (DOTS)-based strategy in Center for Disease Control (CDC) public health clinics in 13 provinces covering half the population, requiring hospitals to refer suspected TB patients to the CDC system. In the early 2000s, the DOTS program was expanded nationwide and an Internet-based disease reporting system was introduced [8-11], further increasing referrals from the hospital to the CDC system. By 2010 it was estimated that approximately 80% of all TB patients were confirmed and treated within the CDC system [8,9], where the treatment success rate was estimated to be 85% [11].

Simultaneously with the DOTS ramp-up, using serial nationwide prevalence surveys, China documented a 65% reduction in prevalence of smear-positive TB over the period from 1990 to 2010 [6-9]. These gains enabled China to achieve the 2015 global TB control target of halving TB prevalence five years before the target date [8]. In spite of these gains, China still has nearly a million incident TB cases annually and nearly a quarter of the world’s multi-drug-resistant (MDR) TB cases [1].

Given the success of the DOTS ramp-up in the CDC public health clinics, it is possible that simply maintaining this level of TB control will enable China to reach the 2035 global targets. However, as the risk of infection has fallen, the burden of disease has shifted to older population groups, which is reinforced by a growing elderly population in China [12-14]. Thus, reactivation disease poses a growing challenge to TB control, as has been observed in Hong Kong [15]. This could limit the continued impact of the DOTS strategy, which does not specifically address reactivation disease in the short term.

To assess the potential impact of reactivation disease and the aging demographics in China, we developed an individual-based transmission model of TB in the Chinese population, explicitly considering the changing demographics of China and the changes in the patient treatment seeking pathway over the last 20 years. This individual-based model builds on the modeling efforts of previous groups [14,16-23], enabling flexibility in the use of available demographics data and changing pathways to care over time.

Using realistic age structure and fitting to age-dependent prevalence data, we independently estimate the contribution of reactivation disease as a driver of TB incidence. In this context, we explore the impact of a basis set of interventions, based on available or soon-to-be-available tools. This basis set represents a set of independent interventions which, in combination, represent all intervention strategies that could possibly be implemented using the available or soon-to-be-available tools. All interventions were modeled optimistically with 100% nationwide coverage starting in 2015. These interventions were parameterized based on what was deemed feasible to implement in the Chinese health care system; they include:

1. increased access to care, by increasing patient referrals from private hospitals to the CDC such that all TB patients are confirmed and treated in the CDC.
2. reduced time to treatment by reducing provider and diagnostic delay using new diagnostics and/or streamlining the diagnostic pathway. This has been achieved in smaller pilot studies with varying levels of success and can reduce the time to treatment up to 50% [24-29].
3. increased treatment success within the CDC, using new drugs/drug regimens which would be effective in patients with both drug-sensitive (DS) and MDR TB. Several new drug regimens are currently in clinical trials or have recently been approved, including bedaquiline, the REMox regimen, and the PaMZ regimen, which have the potential to increase the treatment success rate regardless of MDR status [30-35].
4. active case finding in elders > 65 years old, by combining active TB screening with the annual health screening done in this group [36]. Individuals found by active case finding would be provided with care in the CDC health system.
5. preventative therapy in elders > 65 years old, where latent screening of patients is done in combination with the annual health screening done in this age group. Although in reality latent screening would be done in combination with active case finding, we first model preventative therapy alone in order to explore the basis set of interventions. The combination with active case finding is also modeled. We also note that while preventative therapy is included in the list of interventions because it is a currently available tool, it is not deemed feasible in China due to the age of the screened population, and the relative risks of hepatic adverse events in this age group [37,38]. In addition, general population screening is not considered feasible at this time due to the overall scale of the population.

We use the model to estimate the contribution of reactivation disease to overall burden at the current time. We also quantify the impact of maintaining the DOTS strategy or expanding the TB control strategy to include new interventions. From this analysis, we discuss the feasibility of China reaching the 2035 global targets using existing tools for TB control.

## Methods

**Figure 1:**
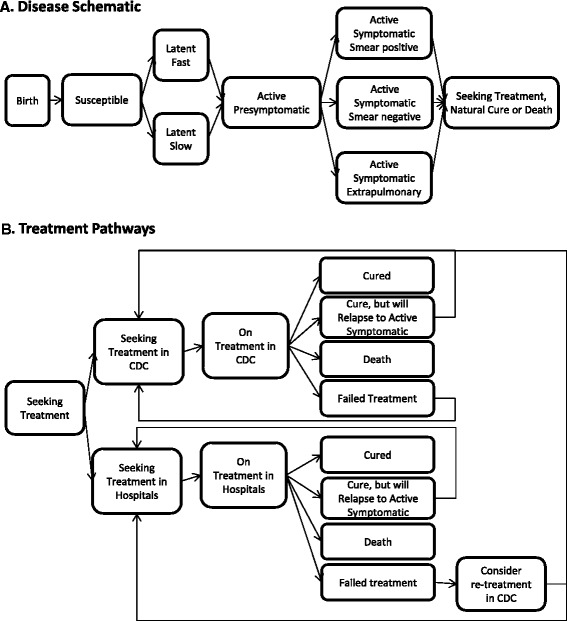
Model and treatment schematic. **A**. Model schematic. Individuals are born healthy and may subsequently acquire latent TB infections through transmission. Disease progresses through latent disease, binned into latent fast or latent slow, through an active presymptomatic phase, and to active symptomatic disease. Individuals in the active presymptomatic and active symptomatic phases are infectious (excluding those with extrapulmonary TB). At the start of active disease, individuals may seek treatment. Individuals may die from non-disease mortality at any phase, but disease mortality only occurs in the active symptomatic phase. **B**. Treatment pathways. Individuals seek treatment either in the CDC or in private hospitals. Once on treatment, they can either be cured, relapse, fail, or die during treatment. Individuals who fail treatment in hospitals can seek retreatment in the CDC or again seek treatment in hospitals. See Additional file 1 for additional details on how the disease progression and treatment pathways were handled in the model.

**Figure 2:**
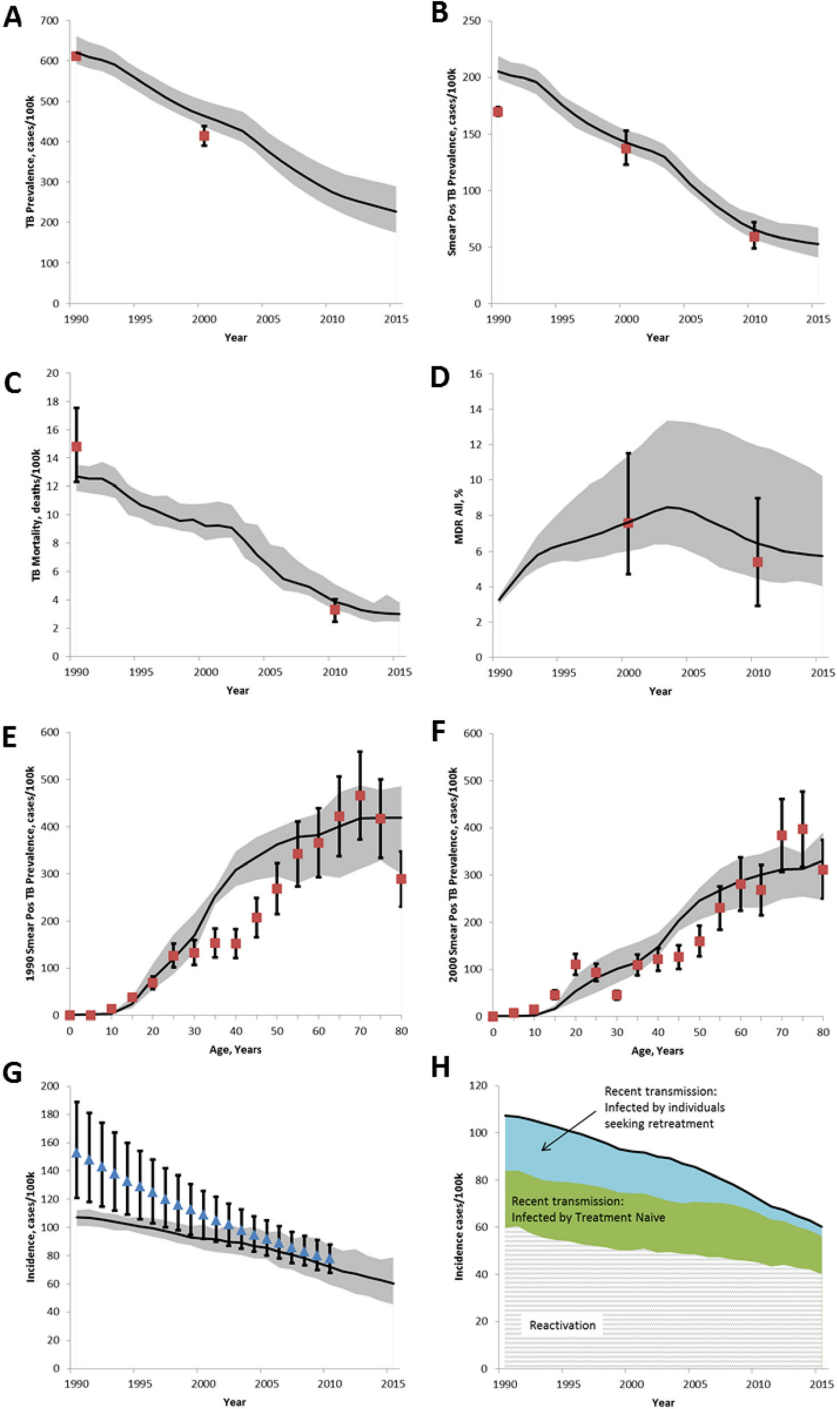
Model calibration to available data. **A**. TB Prevalence, data from [9]. **B**. Smear positive TB prevalence, data from [9]. **C**. Mortality, data from [97]. **D**. MDR, data from [77,78]. **E**-**F**. Age-dependent smear positive prevalence in 1990 and 2000 [77,78]. **G**. Incidence, WHO estimate [1] not used for calibration but shown for comparison. **H**. Breakdown of sources of incidence, model estimate. Solid black line is mean of model output, gray shaded area is 95% credible interval including both parameter and stochastic uncertainties. Red squares represent data (as cited) with reported 95% credible interval.

The present study utilizes the Disease Transmission Kernel (DTK) model developed by the Institute for Disease Modeling group at Intellectual Ventures. The model and all necessary input files are available by request at the Institute for Disease Modeling website [39]. Additional file 1 details the model structure, assumptions, and a complete list of model inputs. The model schematic is included in Figure 1. The fit of the mean trajectory to the data during the calibration time period is shown in Figure 2.

### Model population

We use a simulated population of 500,000 individuals in 1990 that grows to 720,000 by 2035. This represents a 0.05% sampling of the true population size in China. The absolute population value was chosen to reflect available computational resources. The change in population is driven by the UN Population database estimates for medium fertility and age-disaggregated non-TB mortality [40]. The overall population growth is shown in Additional file 1: Figure S1.

### Model initialization

We use a 100-year burn-in period before the calibration period, which is from 1990–2012. The purpose of the burn-in period is to enable simulated individuals to have age-appropriate TB exposure by the end of the burn-in period and the beginning of the model calibration period. At the beginning of the simulation, the population is initialized with 5% prevalence of latent TB with no treatment available until 40 years before the start of the calibration period, at which time hospital treatment is made available to 90% of patients (the remaining 10% of patients have no access to care). The burn-in period is continued for another 40 years until the start of the calibration period (1990), at which time the prevalence and incidence of TB are near steady state, reflecting the observed relative stability in TB prevalence measured in 1990 and 2000 in provinces which did not have any TB-specific interventions during the 1990s (these provinces received DOTS-based treatment during the 2000s) [4-11]. Thus, the absolute value of the population TB prevalence at the beginning of the burn-in period did not affect the steady state equilibrium which defined the start of the calibration period. We allow the acquisition of MDR beginning five years before the end of the burn-in period, reflecting the introduction of rifampin in 1985 and the known non-steady state growth of MDR in 1990 [41]. In order to model the two-step expansion of DOTS [4-11] and because the rate of interprovincial migration is less than 5% [42], we model two distinct homogeneous mixing pools (representing the provinces that received DOTS in the 1990s and 2000s, respectively) with the population evenly divided between them. Acknowledging the reality and challenge of parameterizing the age-stratified population mixing and age-stratified migration rates at the country level, we assume transmission is age-independent in this analysis.

### TB natural history

We stratify disease progression based on age group (adults > 15 and children < 15 years old) and hold constant all disease parameters throughout the simulation, to clearly delineate the role of the aging population structure in China. We did not further stratify the disease parameters in elders [43]. The summary list of relevant parameters is included in Table 1. Individuals are born uninfected and all individuals are vaccinated with bacille Calmette-Guerin (BCG), which reduces the probability of infection by 50% for 10 years [44-47]. We also include an age-dependent reduced susceptibility to infection of 40% from age 2–10, reflecting the BCG-independent reduced susceptibility to TB during the “safe school years” in this age group [48,49]. Once infected with latent TB, individuals are binned into either a latent fast or latent slow stage, representing the distribution of rates with which individuals may progress from latent to active disease. For individuals with the slow rate of progression, this probability of progression is calibrated. Individuals in the latent stage are not infectious and no additional mortality is associated with this phase [50]. Individuals binned into the latent fast stage progress to active symptomatic with a median time of 4 months [51,52]. We calibrate the rate of reactivation in latently infected individuals binned into the latent slow group. Reflecting the spectrum of disease from latent to active disease [53-59], individuals progress from latent disease to an active presymptomatic phase before progressing to a symptomatic phase. The presymptomatic phase represents a period of reduced infectivity where patients may have objective signs of TB (such as increased cough) but lack subjective symptoms (that is, they do not notice their increased cough). The duration of this stage was estimated to be 1 year [56-59]. No additional mortality is associated with this stage.

**Table 1:** **Key model input parameters**

**Parameter**	Range*	**Source**
**Latent phase**		
Proportion of latent disease that is binned into latent fast	Range used in calibration	**This parameter is calibrated** [73,74,98-102]
	Adults: 0.05-0.35	
	Children: set to 1/3 of adult value	
Time to progress from latent fast to presymptomatic active disease	Exponentially distributed	[51,52]
	Mean: 0.5 year	
Time to progress from latent slow to active presymptomatic disease	Exponentially distributed	**This parameter is calibrated** [51,52,55,74,99]
	Range: 5-10% of latent slow reactivate in their lifetime	
Relative reduction in susceptibility to reinfection due to prior exposure to TB	0.65	[74,76,103]
**Active phase**		
Time to progress from active presymptomatic to active symptomatic	Exponentially distributed	[53-59]
	Mean: 1 year	
Proportion of incident active symptomatic TB that is smear positive	Children: 0.15	[48,60,61]
	Adult: 0.585	
Proportion of incident active symptomatic TB that is smear negative	Children: 0.55	[48,60,61]
	Adult: 0.315	
Proportion of incident active symptomatic TB that is extrapulmonary	Children: 0.40	[60,104-107]
	Adult: 0.1	
Duration of active symptomatic disease (until disease resolution) if no treatment is available	Mean 5.5 years	[65]
Disease resolution in smear-positive patients, proportion self-cure	0.3	[65]
Disease resolution in smear-positive patients, proportion death	0.7	[65]
Disease resolution in smear-negative and extrapulmonary patients, proportion self-cure	0.8	[65]
Disease resolution in smear-negative and extrapulmonary patients, proportion mortality	0.2	[65]
**Infectiousness**		
Infectiousness during latent phase	0	[50]
Infectiousness of active presymptomatic TB, relative to smear positive	0.1	[53-59]
Infectiousness of extrapulmonary TB, relative to smear positive	0	[108]
Infectiousness of smear-negative TB, relative to smear positive	0.15	[62-64]
Infectiousness during active symptomatic, smear-positive (contact rate) number of new infections per year	Range used in calibration: 1–7.5	**This parameter is calibrated** [47,82,103,109]
Relative fitness of MDR strain	1	[66-70]
**Treatment seeking**		
Rate of seeking treatment	Exponentially distributed	[24-28,71]
	Mean: 4 months	
Rate of seeking retreatment (hospital)	Exponentially distributed	Assumption informed by expert opinion from Chinese CDC
	Mean: 22 months	
Rate of seeking retreatment (CDC)	Exponentially distributed	Assumption informed by expert opinion from Chinese CDC
	Mean: 4 months	
Relapsers - time from completion of treatment until relapse	Exponentially distributed	[76]
	Mean: 9 months	

*Parameter ranges were derived from the literature and chosen to be consistent with prior TB modeling work and expert opinion for the available health care system in China. Values are the same for adults and children unless otherwise specified.

Active symptomatic disease is split into three clinically defined states: smear-positive pulmonary, smear-negative pulmonary, and extrapulmonary. Smear-positive disease is more likely in adults (65% of all active symptomatic cases) but represents a smaller fraction of active disease in children [35,48,60,61]. Only pulmonary forms are infectious, with smear-negative disease being only 15% as infectious as smear-positive disease and extrapulmonary disease being non-infectious [62-64]. We calibrated the contact rate, the number of individuals who would be infected by an untreated individual with smear-positive disease. The duration of symptomatic disease has a mean of 5.5 years if no treatment is available [65] and is not modeled as variable with clinical presentation. At the end of the symptomatic disease duration, a smear-positive infection will either self-resolve or result in TB-related mortality [65]. The relative ratio of natural recovery to TB-related mortality varies with clinical presentation and does not vary with age. In smear-negative patients and extrapulmonary patients, the disease can either self-resolve or result in TB-related mortality [65].

### Acquired and transmitted MDR TB

MDR TB and DS TB are independently tracked in the model. We do not track acquisition of resistance to individual drugs or further resistance on top of MDR (that is, extensively drug-resistant (XDR) TB), as these were not expected to have a significant effect on our analysis given the relatively small contribution of MDR to overall incidence. MDR TB can be acquired during treatment for DS TB, occurring at the rate specified in the section on TB treatment: treatment outcomes*.* We assume that the MDR strain is 85% as fit as the DS strain [66-70].

### TB treatment: pathways to care

Two treatment pathways are modeled: the private hospital system and the CDC system with its public health TB clinics [8,11], as shown in Figure 1B. Parameterization of the time to treatment and treatment outcomes was based on expert opinion from the Chinese CDC.

Once an individual enters the active symptomatic phase, they start seeking treatment with a median duration of 4 months [24-28,71]. We do not model provider visits which result in no treatment or the time for patient referral from hospitals to the public health system, so the time to treatment encompasses the overall time of treatment seeking until the start of treatment, including patient delay, diagnostic delay, and provider delay. Within the CDC, the sensitivity of diagnosis, regardless of smear status, is thought to be over 95%, using a combination of symptom screening, X-ray, and ultimately culture [72]. Because of this high sensitivity, we do not disaggregate the many levels of the diagnostic pathway, as this was not expected to have a significant impact on our analysis of the role of reactivation. Thus, at the time of treatment initiation, all patients who are TB positive are provided with treatment.

We model only the provision of first- and second-line treatment, and do not explicitly model each individual drug or the adherence patterns to each drug (see TB treatment: treatment outcomes). Most patients receive first-line treatment. Reflecting the current level of MDR testing and provision of second-line treatment, only 1.3% of MDR-positive, smear-positive patients within the CDC treatment pathway receive second-line treatment. Although this proportion is likely slightly higher in retreatment patients and there may be additional time delay associated with MDR testing, we do not explicitly model this, as it is not expected to have a large effect on our analysis of the baseline or new interventions. The treatment outcomes of MDR patients receiving first- or second-line treatment are listed in Additional file 1: Table S4.

At the end of the treatment duration, those who are cured return to the susceptible pool, with a 65% reduced susceptibility to reinfection [73-75]. Individuals who are initially thought to be cured but will ultimately relapse are tracked separately from true cure. These individuals progress through a latent non-infectious phase having a median duration of 9 months [76], before progressing directly to the active symptomatic phase. At the start of the active symptomatic phase, they seek treatment again. Those who fail a treatment reseek care with a rate dependent on which health system they received their most recent treatment - within the CDC the median time to retreatment is 3 months, reflecting better follow-up in the CDC. If they remain in the hospital system, the median time to retreatment is set at 22 months. Individuals who failed treatment in the hospital sector have a 20% probability of shifting to the CDC for retreatment. These values were set based on expert opinion from the Chinese CDC and to achieve a parsimonious fit with the known data on the proportion of new and retreatment patients within the CDC and hospital systems.

### TB treatment: treatment outcomes

Treatment within the CDC system is generally of higher quality than that available in private hospitals. We model only DOTS and second-line combination therapy, and do not disaggregate individual drugs. We also do not model individual treatment adherence patterns, instead including those who drop out as treatment failures. All treatment outcomes were based on data available from the Chinese National TB Control Program and expert opinion [4-7].

In the hospital system, treatment outcomes were set as follows: 55% have long-term cure, 26% fail during treatment, 11% were initially cured but then relapse, and 8% die during treatment. Among those who fail or relapse, there is a 10% probability of developing MDR. In the CDC system, treatment outcomes were set as follows: 82% have long-term cure, 9% fail during treatment, 9% were initially cured but then relapse, and 1% die during treatment. Those who fail or relapse have a 2% probability of developing MDR. These treatment outcomes were slightly poorer if patients were treatment experienced, and are detailed in Additional file 1: Table S3. The parameterization for new treatment based on expected treatment outcomes using new drugs is also described in Additional file 1: Table S3.

MDR patients who received DOTS (that is, due to lack of MDR diagnosis) had treatment outcomes set as follows: 35% have long-term cure, 40% fail during treatment, 10% were initially cured but then relapse, and 20% die during treatment. For the small portion of MDR patients who received second-line treatment in the CDC, the treatment outcomes were slightly higher: 60% have long-term cure, 15% fail during treatment, 10% were initially cured but then relapse, and 15% die during treatment.

### TB treatment: DOTS ramp -up and the shifting access to care

During the calibration period from 1990–2012, we model the historical ramp-up of DOTS according to historically observed patterns [4-11]. During the 1990s, DOTS was implemented in the CDC system through public health clinics in 13 provinces covering half of China’s population, and subsequently expanded nationwide during the 2000s. Treatment within the CDC system was generally of higher quality than that available in private hospitals. (see TB treatment: treatment outcomes). Each of the DOTS ramp-ups was modeled as a linear expansion occurring over three years. From 1992–1995, in the provinces where DOTS expansion occurred in the 1990s, the proportion of patients who did not receive care was reduced from 10% to 5%, and among patients who received care, 60% of them were shifted from the hospital to the CDC. In 2002–2005, changes in the treatment pathways were expanded to the entire country. Country-wide, the proportion of patients with no access to care was reduced to 5%. Of those who did receive care, 80% of patients were shifted from the hospital to the CDC. These transitions are described in Additional file 1: Table S1 and S2.

#### Calibration

The simulation is calibrated to the TB burden (age-dependent prevalence, smear-positive prevalence, and overall prevalence) in China from 1990–2010 as estimated by the Ministry of Health prevalence surveys done in 1990, 2000, and 2010. We also calibrate to the percentage of MDR in new and retreatment patients (survey done in 2007) and the estimated percentage of MDR in all patients (estimated by the Ministry of Health prevalence surveys) [1,7,8,41,77,78].

The model parameters that were calibrated were the contact rate (the infectiousness of a person with smear-positive TB, the average probability of TB transmission per timestep from an infected individual), the fraction of latently infected adults who were classified as fast progressors, and the rate of progression from latent to active in individuals classified as slow progressors. None of these parameters have been directly measured on the country level in China and were deemed most likely to exert the dominant effects on the population-level TB burden and the size of the latent reservoir.

All other parameters were informed by available literature and expert opinion, and were held fixed during the calibration. This reduces the total number of dimensions in the calibration parameter space to a computationally tractable size. This set of fixed parameters includes the total duration of active disease and the relative rate of seeking care in naïve and retreatment patients, which was informed by available information on the time to initial treatment, time to retreatment, and total number of provider visits. While these durations are relevant for analyzing specific interventions which shorten the pathway to care, these values are not expected to have a large effect on our analysis of the relative importance of new transmission and reactivation disease. As our data is restricted to the country-level data, we do not explore individual heterogeneity in the model parameters in this study.

Bayesian calibration using incremental mixture importance sampling [79,80] was employed for parameter space exploration and calibration. Initially, a uniform prior is used for each of the parameters across a plausible parameter range informed by the literature and expert opinion, as detailed in Table 1. We use Latin hypercube sampling to initially explore 1,000 parameter combinations, after which we use the likelihood weighted posterior to select the next round of 60 parameter combinations, for a total of 100 rounds, resulting in 7,000 total parameter combinations sampled from the three-dimensional parameter space.

The likelihood was calculated as the product of the likelihoods for each data point (see Additional file 1 for details on the likelihood calculation and calibration methods). Subsequently, to evaluate the baseline and the impact of new interventions, the likelihood weighted parameter space is resampled 100 times, resulting in a total of 26 unique parameter combinations. These parameter combinations were rerun using 10 random number seeds and averaged together to reduce the stochastic noise. The weighted mean of these parameter combinations thus includes both parameter and stochastic uncertainty. The 26 unique parameter combinations were used to estimate the Bayesian 95% credible interval (see Additional file 1 for details on the calculation).

#### Interventions

All scenarios are modeled optimistically with 100% country-wide implementation in 2015 and impact measured by 2035. This represents the upper limit of impact that could be achieved if these intervention strategies were implemented as described.

1. The first intervention strategy is to increase access to care by shifting the patients currently treated in public hospitals (20% of all patients) to the CDC system, where the quality of treatment is higher. This means 100% of TB patients in China would be treated in a CDC DOTS program.
2. The second intervention strategy involves reducing the time to treatment by reducing provider and diagnostic delay using new diagnostics and/or streamlining the diagnostic pathway. We reduced the time to treatment by 33% for all patients, regardless of which system they used to receive treatment. Treatment naïve patients’ time to treatment was reduced from a median of 128 days to 88 days, for all patients seen in the CDC and hospital system. The time to retreatment was not changed [24-29].
3. Another strategy is increased treatment success within the CDC, using new drugs/drug regimens which would be effective in both DS and MDR patients [30-35]. When given to DS patients, the estimated treatment outcomes are 92% long-term cure, 3.5% fail during treatment, 3.5% are initially cured but then relapse, and 1% die during treatment. This was slightly lowered for MDR and treatment experienced patients. See Additional file 1: Table S3 for the full breakdown of treatment outcome.
4. Active case finding in elders > 65 years old, by combining TB screening with the annual health screening done in this population, is another intervention strategy. Although in reality this would be done throughout the year, this was modeled as a single yearly occurrence for all individuals over age 65, where anyone who was in the active symptomatic phase immediately received treatment from the CDC [36].
5. The final strategy is preventative therapy in elders > 65 years old, where screening of patients is done in combination with active case finding. This was also modeled as a single yearly occurrence for all individuals over age 65. The diagnostic test to identify latently infected individuals was not explicitly modeled. Latent treatment was parameterized as a 9-month regimen with a cure rate of 80% [38]. Individuals who were not cured returned to the latent phase and were eligible to be retreated in subsequent iterations.

## Results and discussion

### Model structure and calibration

We developed a dynamic microsimulation transmission model which tracks individuals from birth to death, including acquisition of latent infection, progression to active disease, treatment seeking behavior, and treatment status. A model schematic is included in Figure 1 illustrating the progression of disease and treatment pathways. Key model inputs are shown in Table 1, and the fit of the mean trajectory to the data during the calibration time period is shown in Figure 2. The decline in incidence over the calibration period is the result of both aging out of the latent reservoir and reduced incidence stemming from infectious individuals seeking retreatment, as high-quality DOTS has lowered the proportion of treated individuals who fail treatment. Additional model outputs are available in Additional file 1.

### Baseline projection

The model estimates that if the status quo in TB diagnosis and treatment is maintained, TB incidence and mortality will decline slowly by 42% (27-59%) and 41% (5-64%, 95% credible interval), respectively, between 2015 and 2035 (Figure 2, Table 2). The combination of continued aging out of the latent reservoir and a low annual risk of infection is consistent with further aging of the epidemic (Additional file 1: Figure S4). MDR is projected to remain relatively stable at below 10% of overall incidence (Additional file 1: Figure S4).

**Table 2:** **Summary of model projections for TB incidence and TB mortality from 2015-2035**

	**2025**	**2025**	**2035**	**2035**
	**Change in active TB incidence**	**Change in mortality**	**Change in active TB incidence**	**Change in mortality**
Status quo	−25% (−15, −39)	−28% (−12, −45)	−42% (−27, −59)	−41% (−5, −64)
DOTS program				
**WHO targets**	**50%**	**75%**	**90%**	**95%**
**Basis set of interventions**				
Expand DOTS to all patients	−28% (−19, −42)	−53% (−36, −71)	−47% (−31, −63)	−65% (−54, −79)
Reduce time to treatment	−29% (−19,- 46)	−33% (−12, −56)	−48% (−35, −66)	−48% (−24, −74)
Improve treatment success	−33% (−24, −44)	−50% (−28, −68)	−49% (−35, −64)	−60% (−43, −77)
Active case finding in elders	−28% (−18, −42)	−42% (−24, −58)	−48% (−34, −64)	−58% (−40, −72)
Preventative therapy in elders	−63% (−53, −76)	−57% (−34, −77)	−79% (−69, −89)	−73% (−60, −75)
**Combinations**				
All feasible interventions*	−42% (−33, −58)	−74% (−64, −85)	−59% (−50, −76)	−83% (−73, −94)
Active case finding and preventative therapy in elders	−63% (−52, −76)	−60% (−44, −79)	−79% (−68, −90)	−75% (−66, −86)
All interventions	−71% (−63, −84)	−85% (−78, −93)	−84% (−78, 93)	−92% (−86, −98)

*All interventions excluding preventative therapy.

Elders considered those > age 65 years old.

95% credible interval shown in parentheses.

**Figure 3:**
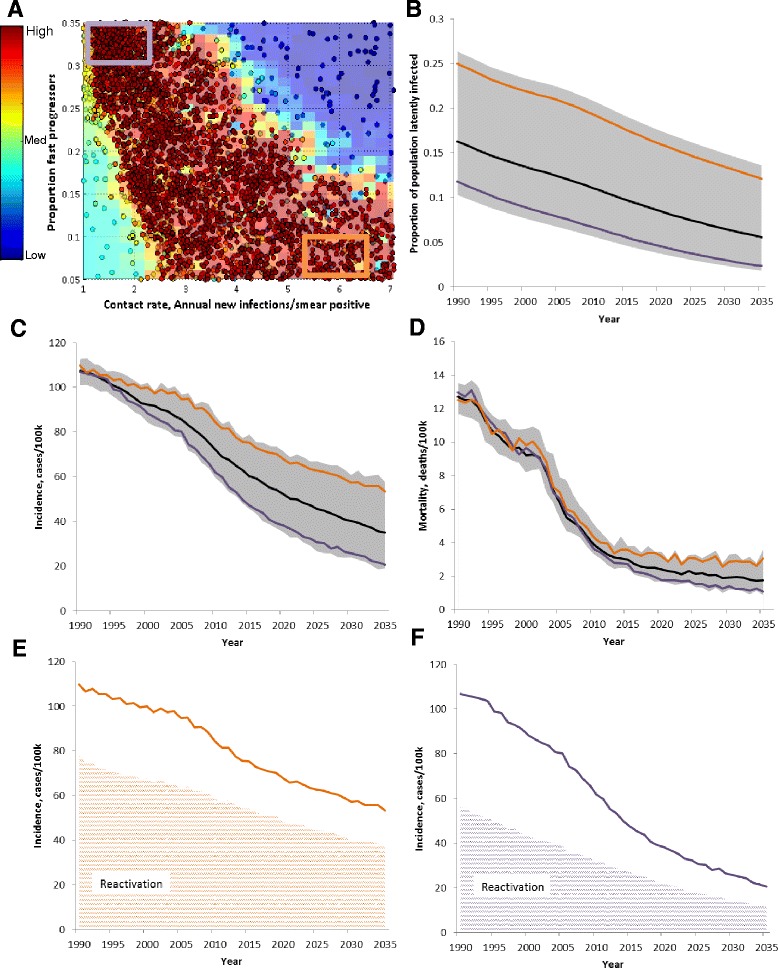
The calibration parameter space and impact on future estimate of TB burden. **A**. The sampled points of the calibration, colored by log-likelihood. Red points have the highest likelihood (see fit in **B**-**F**), while blue points result in trajectories which differ substantially from the data. The orange and purple lines in **B**-**F** are drawn using only sampled calibration points from within the boxes drawn on **A**, where orange represents calibration points with a higher contact rate and lower proportion of fast progressors, while purple represents a lower contact rate and a higher proportion of fast progressors. **B**. Proportion of the population latently infected is higher when a higher contact rate and lower proportion of fast progressors is used. **C**, **E**, **F**. The projected decline in incidence is lower when a higher contact rate is used. The higher absolute incidence is driven by reactivation from the latent reservoir as shown in **E** and **F**. **D**. The trend in mortality follows incidence. Gray shaded area is 95% credible interval.

The baseline projection represents a mean of several points resampled from the calibration parameter space. Notably, there are significant tradeoffs among the calibration parameters that can result in similarly good fits to the data but which estimate different future trends in incidence (Figure 3). Simulations having a higher contact rate and a lower fast progressor fraction (orange curve, based on calibration points within the orange box in Figure 3A) have a larger latent reservoir, and incidence is dominated by slow progressors reactivating from this reservoir. In contrast, simulation which have a lower contact rate and a higher fast progressor fraction (purple curves, based on calibration points within the purple box in Figure 3A) have a smaller latent reservoir; in this parameter space, the total incidence is lower and is projected to decline to a lower level if the DOTS strategy is maintained. The model estimate for the future trend in mortality is consistent with the model estimate in the trend of overall incidence. As variation in the calibration parameters does not affect the individual case fatality rate, simulations that estimate a higher overall incidence also estimate a higher overall mortality.

### Future intervention strategies

**Figure 4:**
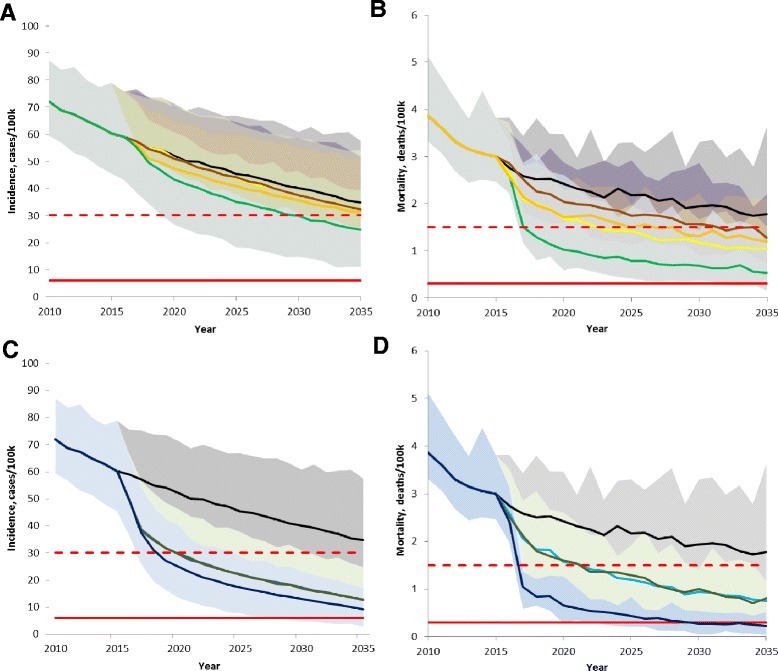
Impact of interventions on TB incidence and mortality from 2010 to 2035. **A**, **B**. None of the feasible interventions, even in combination (bright green), achieve the 2035 incidence or mortality targets. Also shown are the feasible interventions in isolation: baseline (black), expand DOTS (yellow), new drugs (orange), and reduced time to treatment (brown). **C**, **D**. Addition of preventative therapy to the feasible interventions (dark blue line) is likely to nearly reach the 2035 targets for both incidence and mortality. Preventative therapy alone (dark green) and active case finding plus preventative therapy (brown) also shown. The 2025 milestone (red dashed line) and 2035 target (red solid line) are calculated from 2015 model estimated mean value. Shaded area represents 95% credible interval including both parameter and stochastic uncertainties.

All of the modeled interventions are parameterized based on feasibility within the existing health care ecosystem of private hospitals and public CDC clinics. The relative impact of these interventions is described in Table 2 and shown in Figure 4.

1. Increased access to care could be achieved by increasing patient referrals from private hospitals to the CDC. This would increase from 80% to 100% the percentage of TB patients who are confirmed and treated in the CDC clinics, where treatment success rates are higher (see Additional file 1 for detailed tables of the treatment success rates). It would also provide all patients with better follow-up if retreatment is necessary, reducing the infectiousness from treatment experienced individuals. The model estimates that this will result in an estimated reduction in TB incidence and mortality by 47% (31-63%) and 65% (54-79%), respectively, over 20 years. This intervention is the most effective single intervention that is feasible.
2. Reducing the time to treatment shortens the duration of infectiousness in treatment naïve individuals, and could be achieved by using new diagnostics and/or streamlining the diagnostic pathway [24-29]. A one-third reduction in mean time to treatment from 128 days to 88 days for treatment naïve patients in both the hospital and CDC system would result in a limited impact in both TB incidence and mortality compared to baseline.
3. Improving treatment success via more effective drug regimens and better treatment monitoring is the second best intervention. This would be effective for both DS and MDR patients, and reduces the need for retreatment in all patients. By doing so, this intervention also reduces the infectiousness stemming from treatment experienced individuals. The model estimates that this will result in an estimated reduction in TB incidence and mortality by 49% (35-64%) and 60 (43-77%), respectively, over 20 years.
4. Active case finding in elders reduces the time to treatment, primarily for treatment naïve individuals but also affecting those who are treatment experienced. In addition, because it is combined with the annual health screening already done for elders > 65 years old, this intervention would be targeted rather than used in the general population. It is estimated that this would result in a decline in TB incidence and mortality of 48% (34-64%) and 58% (40-72%) over 20 years.
5. Preventative therapy in elders > 65 years old would be the most effective single intervention if it could be made feasible in this age group by addressing the relative risks of hepatic adverse events [37,38]. Preventative therapy is modeled as a 9-month treatment with an overall treatment cure rate of 80% [38]. This strategy directly reduces the size of the latent reservoir. In the first few years, the bulk of the latent reservoir is treated, resulting in a rapid decline in incidence from 2015–2025, and reaching the 2025 milestone for incidence decline. However, the subsequent decline in incidence from 2025 to 2035 is not steep enough to reach the 2035 global target.

The combination of all feasible interventions (all interventions except for preventative therapy) has a larger impact than any of the individual feasible interventions alone but still a smaller impact than preventative therapy. Between 2015–2035, implementing all feasible interventions is estimated to result in a 59% (50-76%) and 83% (73-94%) decline in incidence and mortality, nearly achieving both the 2025 milestone for mortality and the 2035 mortality target. With regards to incidence, the steepest decline is seen between 2015–2025, nearly reaching the 2025 milestone, but the total decline from 2015–2035 does not reach the 2035 incidence target. Including preventative therapy with all feasible interventions has the greatest impact of all the modeled interventions, and is likely to enable China to nearly reach the 2035 incidence and mortality global targets. Between 2015–2035, the projected change in incidence and mortality is −84% (78-93%) and −92% (86-98%).

### Intervention impact is sensitive to calibration parameters

**Figure 5:**
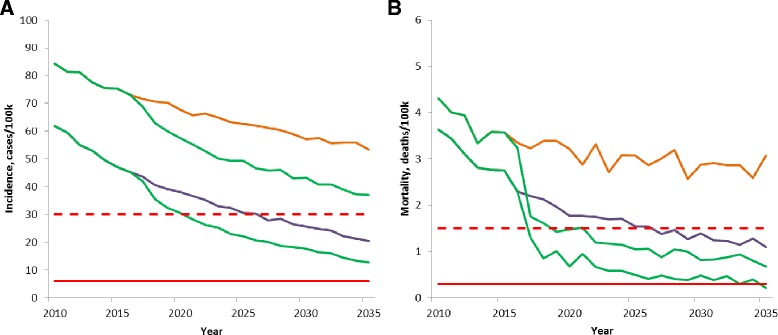
Parameter uncertainty effect on future projection of all feasible interventions. Drawing only from selected areas in parameter space (see Figure 3A), the projection of incidence and mortality are divergent at baseline and with all feasible interventions. **A**. Parameter uncertainty (orange and purple lines) affects future projection of nearing incidence target more than all feasible interventions (green line), including shifting all patients to high-quality care, improving treatment quality, reducing delay, and active case finding. **B**. Implementing all feasible interventions (green line) will result in a dramatic drop in TB mortality, reaching the 2025 milestone and, from some points in parameter space, reaching the 2035 mortality target. The 2035 target (red solid line) is calculated from 2015 model estimated mean value. Orange and purple lines represent the model projection from different areas in parameter space (see Figure 3).

Recognizing that the specific calibration parameters present strong differences in the estimated trend of incidence and mortality (Figure 3), we directly compared the impact of the feasible interventions from different areas in the calibrated parameter space (Figure 5). Comparing model outputs which use the orange and purple boxes in parameter space, the projection of incidence from 2015–2035 is quite divergent, both at baseline and with all feasible interventions. The absolute impact of implementing all feasible interventions is larger if the higher contact rate is used. This is consistent with a higher absolute amount of recent transmission that is driven by the higher contact rate. The estimate for absolute incidence with all feasible interventions given a higher baseline contact rate (green line from orange baseline) is higher than the estimate from baseline at a lower contact rate (purple line). This suggests that in addition to modeling specific interventions, it is necessary to more completely specify the calibrated parameter space.

## Conclusions

The combination of an aging demographic in China and the increasing role of reactivation disease represents a growing challenge to TB control as China considers its post-2015 strategy. We have constructed a mathematical model of TB transmission at the country level in China, taking into account aging of the population and estimating the contribution of reactivation to overall incidence. The nationwide roll-out of the DOTS program reduced the annual risk of infection(ARI) [81,82] by improving treatment outcomes and reducing infectiousness from treatment experienced individuals. Given the high population coverage of DOTS in the CDC public health clinics, we estimate that new transmission is not the major driver of overall TB incidence. Rather, reactivation disease, combined with the growing elderly population, will be the major determinant of the decline in TB incidence and mortality over the next two decades.

Our work shows that if the status quo DOTS strategy is maintained, the TB burden in China will decline but will not reach the 2025 milestones, even if an additional decade is provided. However, additional data are necessary to better specify what the baseline incidence trajectory might be, as projections from different points in the calibration parameter space are divergent over the next 20 years. This could include data on the percentage of the population that is latently infected, stratified by age. The current model estimate for the latent fraction is in line with that estimated in rural areas [83] and in smaller (non-country level) studies, which have primarily focused on city subpopulations or high-risk groups such as health care workers [84]. It is possible that further data collection and analysis in this area can be used to make improved projections of incidence and mortality. Data on the proportion of incidence due to reactivation versus recent transmission could also help to specify the incidence trajectory, though country-specific data in this area is limited and considerable additional data collection would likely be necessary. As shown in this work, this improved specification of the model is equally important to the model estimate of new interventions.

The best single intervention is a system innovation whereby all TB patients would receive their initial treatment in a DOTS program. A combination of all feasible interventions, including expanded access to high-quality care, improved treatment quality, shorter treatment delay, and active case finding in elders will reduce incidence and mortality by 59% (50-76%) and 83% (73-94%), respectively, by 2035. This nearly achieves the 2035 mortality target and suggests that the 2025 incidence milestone can be achieved by 2035 if all feasible interventions are implemented. Further, if preventative therapy can be made feasible in elders, this would be a transformational intervention which is very likely to enable China to reach the 2035 targets.

Overall, our analysis of intervention strategies, selected based on availability of current tools and the structure of the Chinese health care system, suggests that if all feasible interventions are implemented, China may come close to achieving the 2035 mortality target but is unlikely to achieve the 2035 incidence target. Tools aimed at reducing reactivation from the latent reservoir will be critical to quickly reduce incidence in China. This could include a better drug regimen for preventative therapy and/or better monitoring [85,86]. Alternatively, non-TB-specific interventions that might affect the secular trend in TB disease, which were not explicitly modeled in this study, including improved nutrition and better living standards, hold forth the possibility of limiting the rate of reactivation [87-91].

Our model is limited by our assumptions regarding model structure and implementation of new interventions which affect our estimates of the TB burden. First, we assume the disease parameters, including the rate of disease progression and the infectiousness of active disease, remain constant during the entire simulation period. We do not explicitly model how secular trends in transmission related to changing living patterns might have affected the disease parameters [87-91]. Although this would directly influence our results, empiric data in this area are sparse and could either raise or lower our estimate of the role of reactivation disease. A growing population of healthy elders would have a lower likelihood of reactivation, while a growing population of elders living with immune-modulating diseases such as diabetes could result in an overall higher likelihood of reactivation with age [91]. Secular trends in transmission due to changing living conditions, urbanization, and migration could also change the TB transmission rate, directly affecting our estimates of how new interventions might lower TB prevalence. We restrict our analysis in this study to the role of the aging population assuming the disease and transmission parameters remain constant.

Second, we have assumed homogeneous mixing, a simplifying assumption which does not account for spatial differences, non-uniform age-based mixing, and inter-individual heterogeneities in susceptibility and infectivity. Data from the TB prevalence surveys have shown a difference in TB prevalence between rural and urban areas [9], likely due to differences in socioeconomic status, contact patterns, demographic structure, and access to care. Rural-to-urban migrants may face additional delays in care seeking due to geographic and financial restrictions [92,93]. Further, age-dependent mixing is a notable contributor to the age-dependent incidence observed for a variety of respiratory illnesses [94-96], and also varies in urban and rural areas. Our current analysis is restricted to the country level, and does not explicitly model the population heterogeneity which could help or hinder the attainment of the TB control targets. In addition, we have not modeled heterogeneity among individuals and during the course of the infection.

We are optimistic that ongoing data collection in well-instrumented sites will enable better quantification of these unknowns, improving the quality and utility of TB modeling to inform TB control programs. Third, because we have not explicitly specified how these interventions would be operationalized, we do not address the relative cost of the interventions.

Our work suggests that after ramping up DOTS to a high population coverage, reactivation from the latent reservoir plays a growing role in driving incidence, and interventions using existing tools to further reduce new transmission will have a limited impact. To eliminate TB as a public health problem in the Chinese setting, transformative approaches that can limit or prevent reactivation of latent TB infection will likely be needed.

## Additional file

Additional file 1: **Supplementary material describing model structure, calibration and calculation of the credible interval.**

## Abbreviations

ARI: annual risk of infection
BCG: bacilli Calmette-Guerin vaccine
DOTS: directly observed treatment short-course
DS: drug sensitive
DTK: disease transmission kernel
MDR: multi-drug resistant
TB: Tuberculosis
WHO: World Health Organization
XDR: extensively drug resistant
