# Supplementary material for: Tuberculosis control strategies to reach the 2035 global targets in China: the role of changing demographics and reactivation disease
Source: BMC Med. 2015 Apr 21;13:88. doi: 10.1186/s12916-015-0341-4 (PMC4424583; doi:10.1186/s12916-015-0341-4)
Supplement: Supplementary file 1 — Supplementary material describing model structure, calibration and calculation of the credible interval. [file 12916_2015_341_MOESM1_ESM.docx]

**Tuberculosis control strategies to reach the 2035 global targets in China: the role of changing demographics and reactivation disease**

Grace H Huynh, Daniel J Klein, Daniel P Chin, Bradley G Wagner, Philip A Eckhoff, Renzhong Liu, Lixia Wang

**Supporting Information**

We developed a dynamic transmission model of TB in China. The UN Population estimates for crude fertility and age-dependent mortality was input to establish the age demographics of China from 1990-2035. The model scales up DOTS from 1992-2012, and is calibrated to China’s TB burden during this time period. Finally, the calibrated model is run for an additional 20 years to estimate the impact of future interventions (years 2015-2035).

*Model Population*

The simulated population begins with an initial population of 500,000 individuals (representing 0.05% sampling of the true Chinese population for computational purposes), and then is “burned-in” for 100 years using a 5 day timestep to arrive at the appropriate age structure for the population in 1990. During this time, the population is subject to non-TB associated fertility and mortality as prescribed by the UN Population estimates, such that the population size at the end of the burn-in period represents the 1990 demographics. Because data is not available prior to 1950, we initialize the population with the 1950 age distribution and grow the population with a fixed age distribution for the first 60 years of the burn in period. For the last 40 years of burn-in (representing 1950-1990) we directly input the UN Population estimates for crude fertility and age-dependent mortality rates. For years beyond 2015, we use the medium fertility projection for crude fertility and the UN Population estimates for age-dependent mortality [1]. The overall population growth and the age structure of the population are shown in Figure S1.


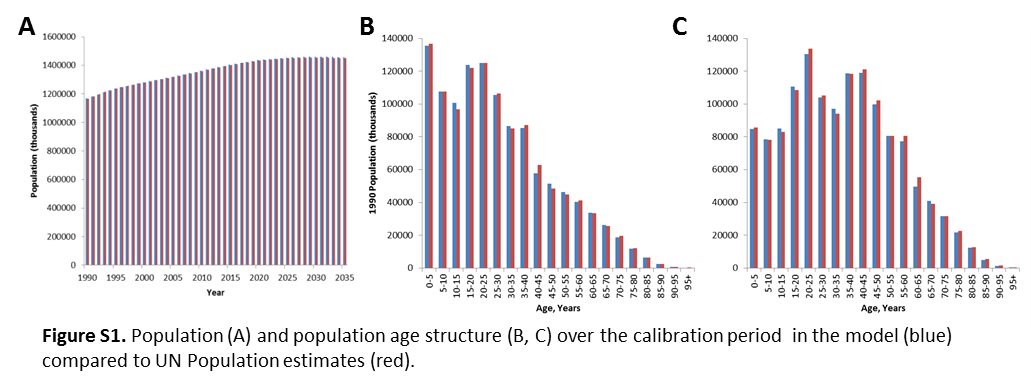


*TB Treatment: DOTS Ramp up and the shifting access to care*

Two treatment pathways are modelled: the private hospital system and Center for Disease Control and Prevention (CDC) system with its public health TB clinics (Wang, 2007; Wang 2009). Parameterization of the time to treatment and treatment outcomes was based on a combination of available survey data and expert opinion from the Chinese CDC, as described in the methods section.

We model the historical ramp up of DOTS according to historically observed patterns (Wang 2007), with a corresponding decrease in the proportion of patients who never access care. This proportion is shown in Table S1.

**Table S1. Increase in the proportion of patients who receive care**

|  | Provinces receiving DOTS ramp up in the 1990s | Provinces receiving DOTS ramp up in the 2000s |
| --- | --- | --- |
| - 1992 (Burn in period) | 0.9 | 0.9 |
| 1992 – 2002 | 0.95 | 0.9 |
| 2002 - 2014 | 0.95 | 0.95 |
|  |  |  |
| All intervention scenarios | 0.95 | 0.95 |

The DOTS ramp up was modelled as a linear expansion occurring over three years. Among patients who did receive care, the proportion of treatment naïve patients which initially accessed the CDC or the Hospital is described in Table S2. From 1992-2002, in the provinces where DOTS expansion occurred in the 1990s, the proportion of patients who have no access to care was reduced from 10% to 5%, and of patients who do get care, 60% of them were shifted from the hospital to the CDC. This change was implemented incrementally over three years. In 2002-2012, changes in the treatment pathways were expanded to the entire country. Country-wide, the proportion of patients with no access to care was reduced to 5%. Of those who did receive care, 80% of patients were shifted from the hospital to the CDC.

**Table S2. Proportion of treatment naïve patients who received care in the Hospital or CDC**

|  | Provinces where DOTS ramp up occurred in the 1990s | Provinces where DOTS ramp up occurred in the 2000s |
| --- | --- | --- |
| - 1992 (Burn in period) | CDC: 0%  Hospital: 100% | CDC: 0%  Hospital: 100% |
| 1992 – 2002 | CDC: 60%  Hospital: 40% | CDC: 0%  Hospital: 100% |
| 2002 - 2015 | CDC: 80%  Hospital:20% | CDC: 0%  Hospital: 100% |

*TB Treatment: Treatment outcomes*

The treatment outcomes for new and retreatment patients receiving DOTS in the hospital and CDC system is shown in Table S3. All treatment outcomes were based on data available from the Chinese National TB Control Program, individual case studies and expert opinion. Among those who fail or relapse in private hospitals, there is a10% probability of acquiring MDR, while the acquisition probability is 2% in the CDC system. The parameterization for new treatment, based on expected treatment outcomes using new drugs is also described in Table 6. These treatment outcomes were based on data available from the Chinese National TB Control Program, expert opinion, and an optimistic outlook based on preliminary data of the effectiveness of new drugs soon to be available (Lienhardt 2010, Diacon 2012, Gillespie 2014, Diacon 2014, Gler 2012, Jindani 2014) . The treatment duration within the hospitals is estimated to be approximately 90 days, reflecting the typical practice in hospitals where TB patients are hospitalized for 2-3 months treatment and subsequently discharged, after which patients typically fail to continue therapy.

**Table S3. Treatment outcome for new and retreatment patients receiving first line drugs, by health care sector.**

|  | New patients | | | | Retreatment | | |
| --- | --- | --- | --- | --- | --- | --- | --- |
|  |  | Hospital | CDC | New drugs | Hospital | CDC | New drugs |
| DS | Treatment Duration | 90 days | 180 days | 120 days | 90 days | 180 days | 120 days |
|  | Cure | 0.55 | 0.82 | 0.92 | 0.55 | 0.75 | 0.9 |
|  | Relapse | 0.11 | 0.08 | 0.035 | 0.11 | 0.01 | 0.045 |
|  | Mortality | 0.08 | 0.01 | 0.01 | 0.08 | 0.01 | 0.01 |
|  | Failed | 0.26 | 0.09 | 0.035 | 0.26 | 0.14 | 0.045 |
|  |  |  |  |  |  |  |  |
| MDR | Treatment Duration | 90 days | 180 days | 180 days | 90 days | 180 days | 180 days |
|  | Cure | 0.1 | 0.35 | 0.85 | 0.1 | 0.35 | 0.82 |
|  | Relapse | 0.15 | 0.1 | 0.06 | 0.15 | 0.1 | 0.07 |
|  | Mortality | 0.25 | 0.2 | 0.03 | 0.25 | 0.2 | 0.04 |
|  | Failed | 0.5 | 0.35 | 0.06 | 0.5 | 0.35 | 0.07 |

*MDR TB*

MDR TB and DS TB are independently tracked in the model. We do not track acquisition of resistance to individual drugs or further resistance on top of MDR (ie XDR), as these were not expected to have a significant effect on our analysis given the relatively small contribution of MDR to overall incidence. MDR TB can be acquired during treatment for DS TB, occurring at the rate specified in *TB Treatment: Treatment outcomes.* Acquisition of MDR is counted towards incidence of MDR TB at the time of MDR acquisition. Transmission of MDR is due to contact from an infectious MDR TB individual to a susceptible individual. Incidence of transmission generated MDR TB is counted towards incident MDR at the time of disease activation. We assume that the MDR strain is 85% as fit as the DS strain, and transmission of MDR is tracked simultaneously with DS TB (Cohen 2004, Dye, Espinal, Borrell, Cohen 2003). For each individual with MDR, the model tracks whether they acquired MDR or were infected with transmitted MDR. At the time all individuals present for treatment (either as treatment naïve or treatment experienced) they are counted in the fraction of new and retreatment cases which are MDR. The treatment outcome for MDR patients who receive second line drugs is listed in Table S4. These treatment outcomes were based on data available from the Chinese National TB Control Program, and expert opinion.

**Table S4. Treatment outcome for new and retreatment patients receiving second line drugs.**

|  | Hospital | CDC |
| --- | --- | --- |
| Proportion MDR patients who receive second line drugs | 0% | 1.5% |
| Treatment Duration | 270 days | 270 days |
| Cure | 0.6 | 0.6 |
| Relapse | 0.1 | 0.1 |
| Mortality | 0.15 | 0.15 |
| Failed | 0.15 | 0.15 |

*Calibration*

The simulation is calibrated to the TB burden (age dependent prevalence, smear positive prevalence, and overall prevalence) in China from 1990-2010 as estimated by the Ministry of Health prevalence surveys done in 1990, 2000, and 2010. We also calibrate to the percentage of MDR in new and retreatment patients (survey done in 2007) and the estimated percentage MDR in all patients (estimated by the Ministry of Health prevalence surveys). We also calibrate to the IHME estimate of mortality in this time period. The full list of calibration data is shown in Table S5.

**Table S5. List of calibration data**

| Likelihood Component Index | Calibration Data | Year and Value (/100,000 unless otherwise noted,)  [95% CI, estimated by data source unless otherwise noted] | | | | Source |
| --- | --- | --- | --- | --- | --- | --- |
| 1 | **Prevalence, country-wide** | 1990: 619 [603-619] 2000: 414 [390-439]  1990: 442 [417-469] | | | Wang 2014 | |
| 2 | **Smear positive prevalence, country-wide** | 1990: 170 [166-174] 2000: 137 [123-153]  1990: 59 [49-72] | | | Wang 2014 | |
| 3 | **Smear positive Prevalence, provinces which implemented DOTS in the 1990s** | 1990: 176 [170-182] 2000: 113 [96-133]  1990: 63 [50-80] | | | Wang 2014 | |
| 4 | **Smear positive Prevalence, provinces which implemented DOTS in the 2000s** | 1990: 180 [174-186] 2000: 174 [151-201]  1990: 59 [43-79] | | | Wang 2014 | |
| 5 | **Age dependent smear positive prevalence, country-wide** | Year | Age (years) | Value (/100,000) | Ministry of Health, 1990  Ministry of Health, 2000 | |
|  |  | 1990 | 0-5 | 0 |  |  |
|  |  | 1990 | 5-10 | 0 |  |  |
|  |  | 1990 | 10-15 | 14 |  |  |
|  |  | 1990 | 15-20 | 38 |  |  |
|  |  | 1990 | 20-25 | 69 |  |  |
|  |  | 1990 | 25-30 | 127 |  |  |
|  |  | 1990 | 30-35 | 133 |  |  |
|  |  | 1990 | 35-40 | 153 |  |  |
|  |  | 1990 | 40-45 | 152 |  |  |
|  |  | 1990 | 45-50 | 207 |  |  |
|  |  | 1990 | 50-55 | 269 |  |  |
|  |  | 1990 | 55-60 | 342 |  |  |
|  |  | 1990 | 60-65 | 366 |  |  |
|  |  | 1990 | 65-70 | 422 |  |  |
|  |  | 1990 | 70-75 | 466 |  |  |
|  |  | 1990 | 75-80 | 417 |  |  |
|  |  | 1990 | 80-85 | 289 |  |  |
|  |  | 2000 | 0-5 | 0 |  |  |
|  |  | 2000 | 5-10 | 7 |  |  |
|  |  | 2000 | 10-15 | 15 |  |  |
|  |  | 2000 | 15-20 | 46 |  |  |
|  |  | 2000 | 20-25 | 110 |  |  |
|  |  | 2000 | 25-30 | 93 |  |  |
|  |  | 2000 | 30-35 | 45 |  |  |
|  |  | 2000 | 35-40 | 109 |  |  |
|  |  | 2000 | 40-45 | 121 |  |  |
|  |  | 2000 | 45-50 | 126 |  |  |
|  |  | 2000 | 50-55 | 160 |  |  |
|  |  | 2000 | 55-60 | 230 |  |  |
|  |  | 2000 | 60-65 | 281 |  |  |
|  |  | 2000 | 65-70 | 268 |  |  |
|  |  | 2000 | 70-75 | 384 |  |  |
|  |  | 2000 | 75-80 | 397 |  |  |
|  |  | 2000 | 80-85 | 312 |  |  |
|  |  | * 95% CI not available so + 20% of the value was assumed | | |  |  |
| 6 | **Mortality, country-wide** | 1990: 18.1 [13.7-23.2]  2010: 3.3 [2.4-4.0] | | | WHO, Murray 2014 | |
| 7 | **Percent of MDR in all patients** | 2000: 7.6 [4.7-11.5]  2010: 5.4 [2.9-9.0] | | | Ministry of Health, 1990 | |
| 8 | **Percent of MDR in new and retreatment patients** | 2007, new patients: 5.7 [4.5-7]  2007, retreatment patients: 25.6 [21.5-29.8] | | | Zhao 2007 | |

Incremental Mixture Importance Sampling (IMIS) (Raftery, Steele) was employed to obtain a set of model parameters consistent with observed data. Three key model parameters were calibrated using this process, see Table 1 for a list of these parameters and their prior distributions.

On the first iteration of IMIS,
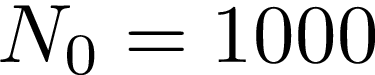
 parameter combinations were sampled from the prior distribution, *
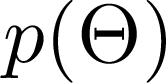
,* using Latin Hypercube Sampling,


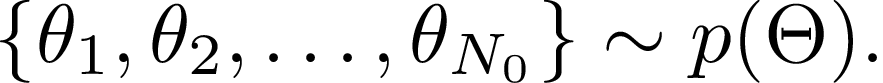


The total likelihood of each parameter configuration,
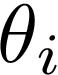
, given the data D, was computed as the product of the
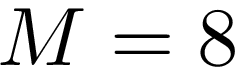
 likelihood components listed in Table 1,


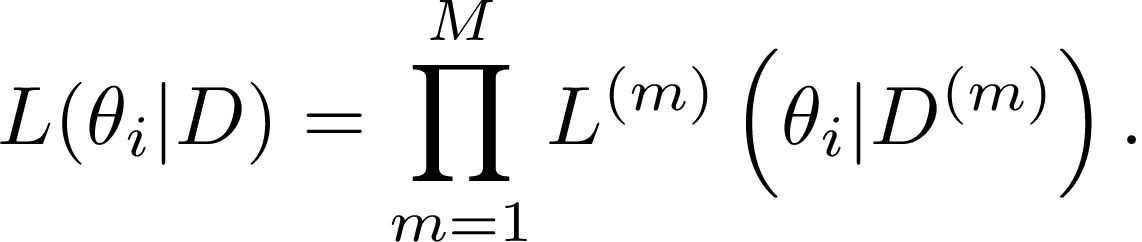


Each likelihood component was computed as the product of
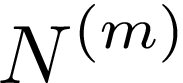
 data points in a subset of *D*,


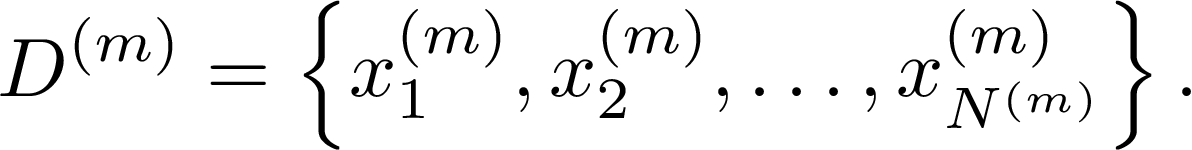


For each parameter configuration,
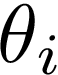
, we ran the TB model and computed the simulated value of each data point,


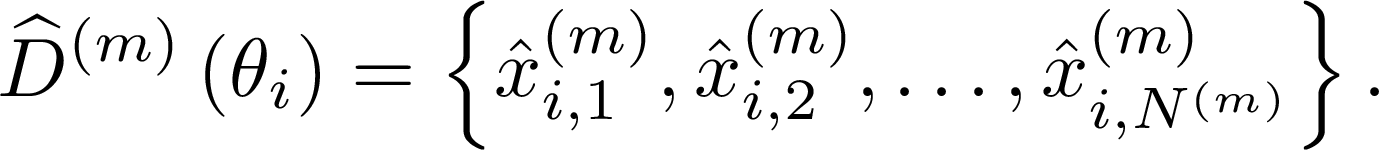


For example, the first component (m=1) in Table 1 is the country-wide prevalence. This component consists of
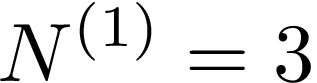
 data points corresponding to the prevalence in years 1990, 2000, and 2010. We ran the model for each parameter combination and computed the prevalence at the appropriate time points.

Individual data points were assumed to be independent from other data points, and have normally distributed errors. The resulting likelihood of each data subset given the model output produced using parameter
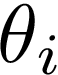
 was computed as


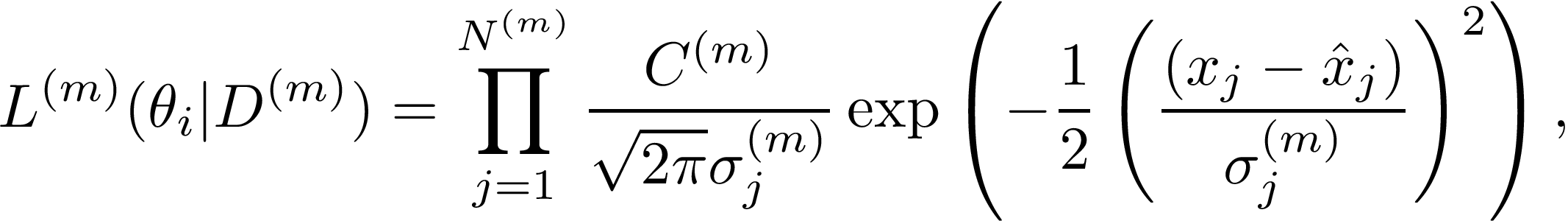


where
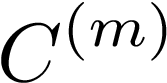
 is a scaling constant to overflow and
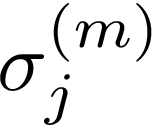
 is the standard deviation of the *j*^th^ data point in data set *m*. These standard deviations were computed from the 95% confidence intervals given in the source data and also provided in Table 1.

After computing all likelihoods, each sample point is assigned an importance weight,


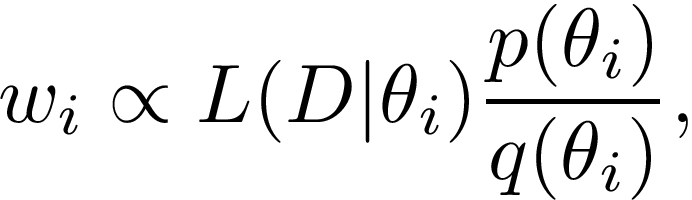


where *q* is the sampling distribution. On the first iteration, the sampling distribution is simply the prior distribution, *p*, so the importance weights are just the likelihoods.

Simulations were run on a 512 core computing cluster. The TB simulation requires 8 cores, so on each iteration of the IMIS algorithm, *B*=60 new runs were simulated, requiring 480 cores total. These runs were drawn from a multivariate normal distribution, centered at the point with the greatest importance weight using a covariance matrix computed from nearby samples. With these new samples, the sampling distribution, *q*, becomes a weighted mixture of prior and the multivariate normal distributions. Refer to (Raftery, Steele) for additional details.

Once a total of 100 iterations were complete, we resampled 100 parameter configurations using the importance weights, resulting in 26 unique parameter configurations.

The dominant interactions between the calibrated parameters are shown in the 2D projections shown in Figure S2.

*
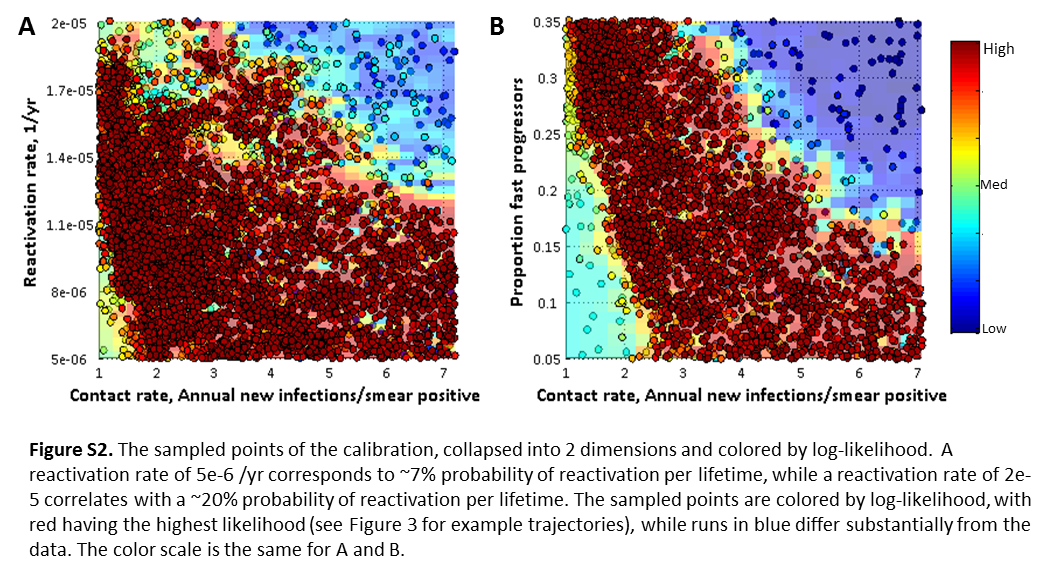
*

*Calculation of Credible Interval*

To evaluate the baseline and the impact of new interventions, the likelihood-weighted parameter space is resampled 100 times, resulting in a total of *P=*26 weighted parameter combinations. The weights range from 1 to 17, and correspond to the number of times each point was selected during posterior resampling. These parameter combinations were re-run using *R=*10 random number seeds and ultimately averaged together to reduce the stochastic noise. The weighted mean of these parameter combinations thus includes both parameter and stochastic uncertainty. We directly compute the 95% credible interval from the weighted sample sets. Results were reported as mean (95% credible interval).

**
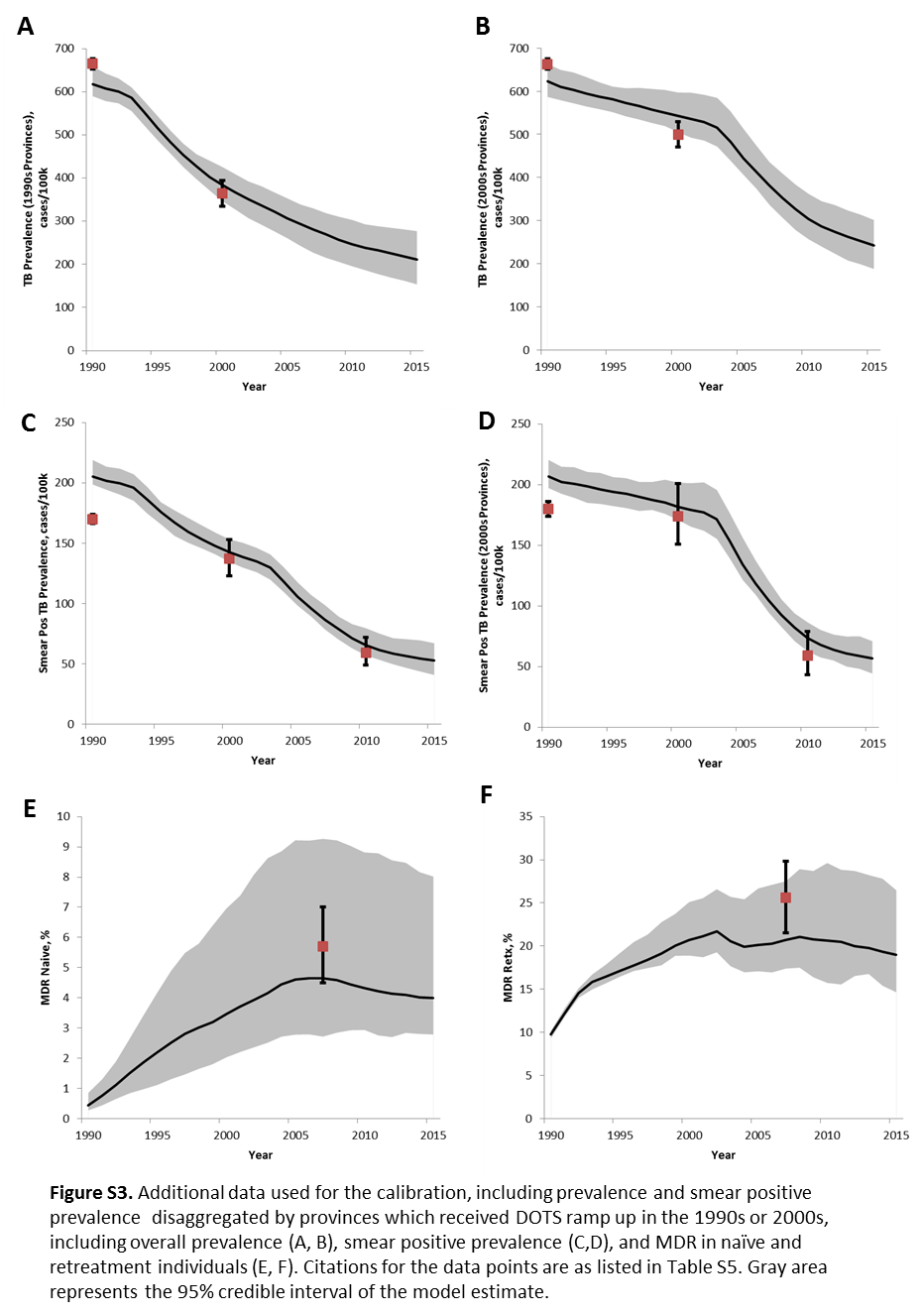
**

**
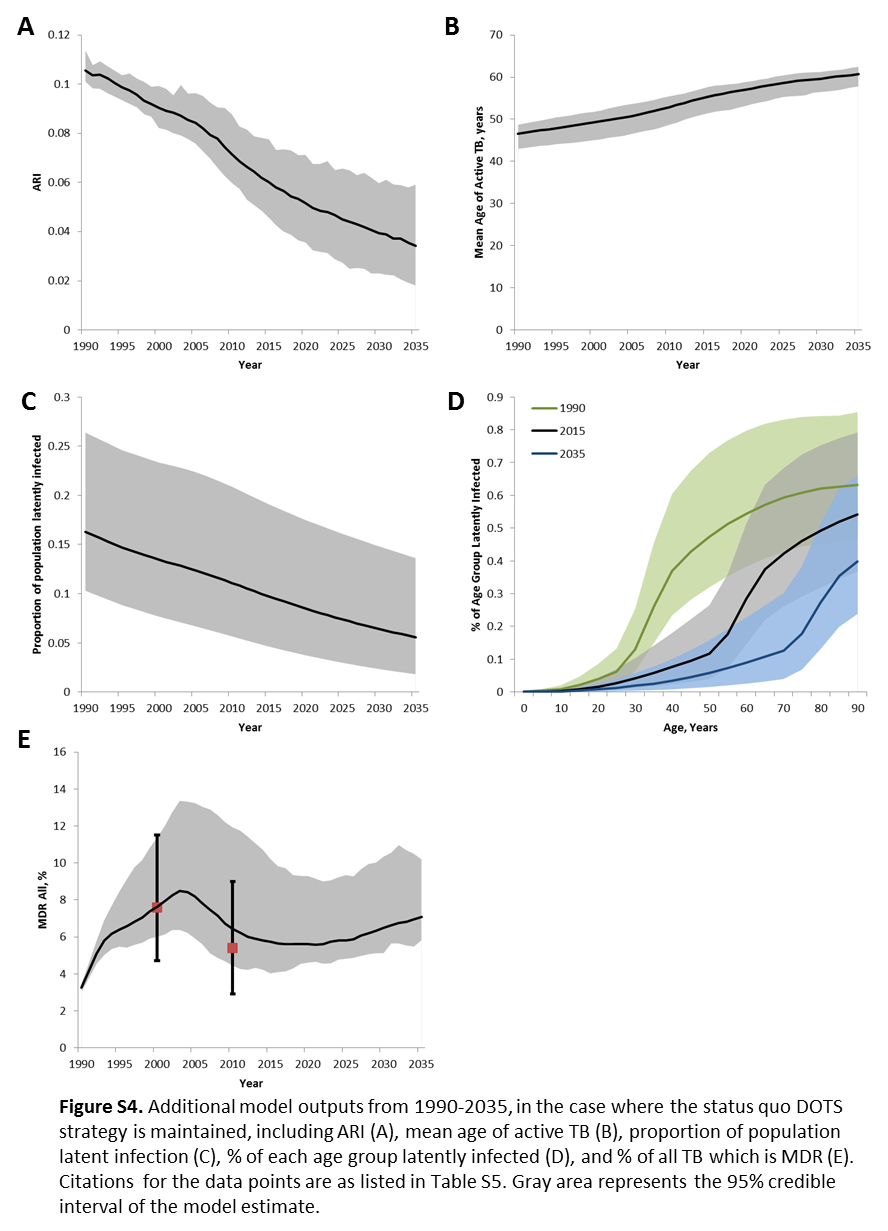
**

**References**

Borrell S, Gagneux S: **Infectiousness, reproductive fitness and evolution of drug-resistant Mycobacterium tuberculosis.** *Int J Tuberc Lung Dis* 2009, **13**(12):1456-1466**.**

China Tuberculosis Control Collaboration: **The effect of tuberculosis control in China.** *Lancet* 2004, **364**(9432):417-422.

Cohen T, Sommers B, Murray M: **The effect of drug resistance on the fitness of Mycobacterium tuberculosis.** *Lancet Inf Dis.* 2003, **3:**13-21.

Cohen T, Murray M: **Modeling epidemics of multidrug-resistant M Tuberculosis of heterogeneous fitness.** *Nature Medicine* 2004**, 10**:1117-1121**.**

Diacon AH, Dawson R, von Groote-Bidlingmaier F, Symons G, Venter A, Donald PR, van Niekerk C, Everitt D, WinterH, Becker P, Mendel CM, Spigelman MK: **14-day bactericidal activity of PA-824, bedaquiliine, pyrazinamide, and moxifloxacin combinations: a randomized trial.** *Lancet* 2012, **380**(9846):986-93.

Diacon AH, Pym A, GrobuschMP, de los Rios JM, Gotuzzo E, Vasilyeva I, Leimane V, Andries K, Bakare N, De Marez T, Haxaire-Theeuwes M, Lounis N, Meyvisch P, De Paepe E, van Heeswijk RP, Dannemann B, TMC207-C208 Study Group: **Multidrug-resistant tuberculosis and culture conversion with bedaquiline.** *NEJM* 2014, **371**(8):723-32.

Dye C, Williams BG, Espinal MA, Raviglione MC: **Erasing the world’s slow stain: strategies to be at multidrug-resistant tuberculosis.** *Science* 2002, **295**:2042-2046.

Espinal MA: **The global situation of MDR-TB.** *Tuberculosis* 2003, **83**(1-3):44-51.

Gillespie SH, Crook AM, McHugh TD, Mendel CM, Meredith SK, Murray SR, Pappas F, Phillips PP, Nunn AJ, REMoxTB Consortium: **Four-month moxifloxacin-based regimens for drug-sensitive tuberculosis**. *NEJM* 2014, **371**(17):1577-1587.

Gler MT, Skripconoka V, Sanchez-Garavito E, Xiao H, Cabrera-Rivero JL, Vargas-Vasquez DE, Gao M, Aqad M, Park SK, Shim TS, Suh GY, Danilovits M, Ogata H, Kurve A, Chang J, Suzuki K, Tupasi T, Koh WJ, Seaworth B, Geiter LJ, Wells CD: **Delamanid for multidrug-resistant pulmonary tuberculosis.** *NEJM* 2012, **366**(23):2151-60.

Jindani A, Harrison TS, Nunn AJ, Phillips PP, Churchyard GJ, Charalambous S, Hatherill M, Geldenhuys H, McIlleron HM, Zvada SP, Mungofa S, Shah NA, Zizhou S, Magweta L, Shepherd J, Nyirenda S, van Dijk JH, Clouting HE, Coleman D, Bateson AL, McHugh TD, Butcher PD, Mitchison DA, RIFAQUIN Trial Team: **High-dose rifapentine with moxifloxacin for pulmonary tuberculosis.** *NEJM* 2014, **371**:1599-1608.

Lienhardt C, Vernon A, Raviglione MC: **New drugs and new regimens for the treatment of tuberculosis: review of the drug development pipeline and implications for national programmes.** *Curr Opin Pulm Med* 2010, **16**:186-193.

Ministry of Public Health of the People’s Republic of China. Nationwide random survey for the epidemiology of tuberculosis in 1990. Beijing: Ministry of Public Health of the People’s Republic of China, 1990.

Ministry of Public Health of the People’s Republic of China. Report on nationwide random survey for the epidemiology of tuberculosis in 2000. Beijing: Ministry of Public Health of the People’s Republic of China, 2000.

Murray CJL et al. **Global, regional, and national incidence and mortality for HIV, tuberculosis and malaria during 1990-2013: a systemic analysis for the Global Burden of Disease Study 2013.** *Lancet* 2014, 384(9947):1005-1070.

Raftery AE, Bao L: **Estimating and Projecting Trends in HIV/AIDS Generalized Epidemics Using Incremental Mixture Importance Sampling.** *Biometrics* 2010, **66**:1162–1173.

Steele RJ, Raftery AE, Emond MJ: **Computing Normalizing Constants for Finite Mixture Models via Incremental Mixture Importance Sampling (IMIS).** *J Comput Graph Stat* 2006, **15**:712–734.

Wang L, Liu J, Chin DP: **Progress in tuberculosis control and the evolving public-health system in China.** *Lancet* 2007, **369**(9562):691-66.

**WHO Global Tuberculosis Report 2014.** [<http://www.who.int/tb/publications/global_report/en/>]

Zhao Y, Xu S, Wang L, Chin DP, Wang S, Jiang G, Xia H, Zhou Y, Li Q, Ou X, Pang Y, Song Y, Zhao B, Zhang H, He G, Guo J, Wang Y: **National survey of drug-resistant tuberculosis in China.** *NEJM* 2012, **366**(23):2161-70.
